# Dragon’s Blood Sap Microencapsulation within Whey Protein Concentrate and Zein Using Electrospraying Assisted by Pressurized Gas Technology

**DOI:** 10.3390/molecules28104137

**Published:** 2023-05-17

**Authors:** Juan David Escobar-García, Cristina Prieto, Maria Pardo-Figuerez, Jose M. Lagaron

**Affiliations:** 1 Research & Development Department, Bioinicia S.L. Calle Algepser 65, 46980 Paterna, Spain; juaesga@gmail.com (J.D.E.-G.); mpardo@iata.csic.es (M.P.-F.); 2 Novel Materials and Nanotechnology Group, Institute of Agrochemistry and Food Technology (IATA), Spanish Council for Scientific Research (CSIC), Calle Catedrático Agustín Escardino Benlloch 7, 46980 Paterna, Spain

**Keywords:** dragon’s blood sap, encapsulation, microparticles, whey protein concentrate, zein, EAPG

## Abstract

Dragon’s blood sap (DBS) obtained from the bark of Croton lechleri (Müll, Arg.) is a complex herbal remedy of pharmacological interest due to its high content in polyphenols, specifically proanthocyanidins. In this paper, electrospraying assisted by pressurized gas (EAPG) was first compared with freeze-drying to dry natural DBS. Secondly, EAPG was used for the first time to entrap natural DBS at room temperature into two different encapsulation matrices, i.e., whey protein concentrate (WPC) and zein (ZN), using different ratios of encapsulant material: bioactive compound, for instance 2:1 *w*/*w* and 1:1 *w*/*w*. The obtained particles were characterized in terms of morphology, total soluble polyphenolic content (TSP), antioxidant activity, and photo-oxidation stability during the 40 days of the experiment. Regarding the drying process, EAPG produced spherical particles with sizes of 11.38 ± 4.34 µm, whereas freeze-drying produced irregular particles with a broad particle size distribution. However, no significant differences were detected between DBS dried by EAPG or freeze-drying in TSP, antioxidant activity, and photo-oxidation stability, confirming that EAPG is a mild drying process suitable to dry sensitive bioactive compounds. Regarding the encapsulation process, the DBS encapsulated within the WPC produced smooth spherical microparticles, with average sizes of 11.28 ± 4.28 µm and 12.77 ± 4.54 µm for ratios 1:1 *w*/*w* and 2:1 *w*/*w*, respectively. The DBS was also encapsulated into ZN producing rough spherical microparticles, with average sizes of 6.37 ± 1.67 µm and 7.58 ± 2.54 µm for ratios 1:1 *w*/*w* and 2:1 *w*/*w*, respectively. The TSP was not affected during the encapsulation process. However, a slight reduction in antioxidant activity measured by DPPH was observed during encapsulation. An accelerated photo-oxidation test under ultraviolet light confirmed that the encapsulated DBS showed an increased oxidative stability in comparison with the non-encapsulated DBS, with the stability being enhanced for the ratio of 2:1 *w*/*w*. Among the encapsulating materials and according to the ATR-FTIR results, ZN showed increased protection against UV light. The obtained results demonstrate the potential of EAPG technology in the drying or encapsulation of sensitive natural bioactive compounds in a continuous process available at an industrial scale, which could be an alternative to freeze-drying.

## 1. Introduction

Polyphenols are a group of natural compounds with phenolic structural features [1]; which represent a diverse subclass of secondary metabolites present in plants [2]. Polyphenols are divided into several sub-classes, such as phenolic acids, flavonoids (flavonols, flavones, flavanols, anthocyanidins, and isoflavones), stilbenes, and lignans, among others [3,4]. These compounds are well-known for their antioxidant properties [5] and for their ability to protect the organism against reactive oxygen species (ROS) [6], which are responsible of several pathologies such as neurodegenerative, lung, and cardiovascular diseases, diabetes, and atherosclerosis [7,8].

Due to their health benefits, the scientific community and the food, pharmaceutical, and cosmetic industries, among others, have paid special attention to the isolation of phenolic compounds from different raw materials, especially from inexpensive or residual sources such as medicinal plants, fruits, vegetables, industrial by-products, and beverages [9]. In this sense, dragon’s blood sap (DBS) obtained from the bark of Croton lechleri (Müll, Arg.) trees is well-known due to its health benefits and as such, it is widely used in folk medicine due to its antioxidant, antimicrobial, anti-inflammatory, and cicatrizing properties, mainly due to its principal chemical constituents such as proanthocyanidins (>90% dry weight of the resin), taspin, catechin, epigallocatechin, and epicatechin [10]. Up to now, numerous studies have been published dealing with the characterization of its bioactive properties [11,12]. Escobar-García et al. demonstrated that the high antioxidant activity of DBS was susceptible to environmental factors such as the irradiation of UV light [9], which may induce a reduction in its health benefits and consequently limit its commercial potential [13].

To ensure the stability of natural bioactive compounds, a wide variety of delivery systems are available [14]. Among them, one of the most appreciated approaches is the encapsulation of the bioactive compounds, which has garnered a growing interest during the last decade to protect natural bioactive compounds that are highly sensitive to the environmental conditions present during processing and storage, as well as in the upper gastrointestinal tract [15]. Through this technology, the active agents are entrapped within an encapsulating matrix, which protects the bioactive compounds from environmental conditions [16]. However, conventional encapsulation technologies such as spray drying, spray coating, freeze drying (FD), co-extrusion, and coacervation [17,18,19,20,21], among others, present multiple disadvantages. For example, spray drying, despite being available at an industrial scale, requires the use of high temperature, which may affect thermolabile bioactive compounds; freeze drying is an expensive process that produces irregular particles with a big particle size distribution, and it takes place in batch mode; and spray coating, co-extrusion, and coacervation, produce particles with a big particle size. All these factors affect the stability of the bioactive compound and the organoleptic properties of the final product and make the process difficult at an industrial level.

However, novel encapsulation technologies have been developed to overcome these challenges, such as encapsulation based on electrohydrodynamic processes [22]. Concretely, the electrospraying process is based on the action of an external electric field over a polymer solution to produce ultrathin droplets, which after solvent evaporation at room temperature result in nano- or micro-sized particles [23]. Additionally, electrospraying is carried out at room temperature, which reduces the denaturation of bioactives, possesses high encapsulation efficiency, results in a dried powder with reduced particle size, and is versatile in terms of the encapsulating matrices that can be used [24]. Thus, this technology has been used for the encapsulation of polyphenols such as (−)-epigallocatechin gallate (EGCG), tannic acid, and green tea extract, among others, and has resulted in promising results [25,26]. However, this technology presents a main disadvantage related to its poor yield, which is lower than 1 g per day in the majority of case studies [27].

Consequently, novel high-yield technology based on the integration of electrospraying, and gas-driven nebulization was developed to overcome the yield limit of electrospraying technology [28]. This novel high-throughput technology, termed electrospraying assisted by pressurized gas (EAPG), is based on the atomization of the polymer solution by a pneumatic injector using compressed air that nebulizes within a high electric field. By means of this technology, it is possible to obtain a throughput larger than 1 kg/h of dry powder on an industrial scale. During this process, the solvent is evaporated at room temperature in an evaporation chamber, and the encapsulated material is then collected as a free-flowing powder. The potential of this technology has been proven in sensitive materials such as omega-3-rich fish oil, algae oil, eicosapentaenoic-acid-rich oil, and pharmaceutical compounds such as carvedilol and valsartan, protected using diverse encapsulating materials, such as whey protein concentrate (WPC), zein (ZN), maltodextrin, wheat gluten extract, polyvinylpyrrolidone, and hydroxypropyl methyl cellulose [23,29,30,31,32]. However, to the best of our knowledge, no scientific studies have been published dealing with the encapsulation of DBS by electrospraying or EAPG. Therefore, in the present work, EAPG was first compared with freeze-drying to dry natural DBS to prove that it is a mild technology suitable to drying sensitive bioactive compounds. The obtained materials were compared in terms of morphology. Secondly, the EAPG process was used to study the encapsulation of DBS into two food-approved encapsulating matrices, i.e., WPC and ZN. These two proteins were selected as encapsulating matrices due to their effective microencapsulating properties as well as their antioxidant and photo-protective properties [24,28,32]. The obtained DBS microcapsules were characterized in terms of morphology, total soluble polyphenols (TSP), antioxidant activity, and accelerated oxidative stability under photo-oxidation conditions.

## 2. Results and Discussion

### 2.1. Morphology

**Figure 1:**
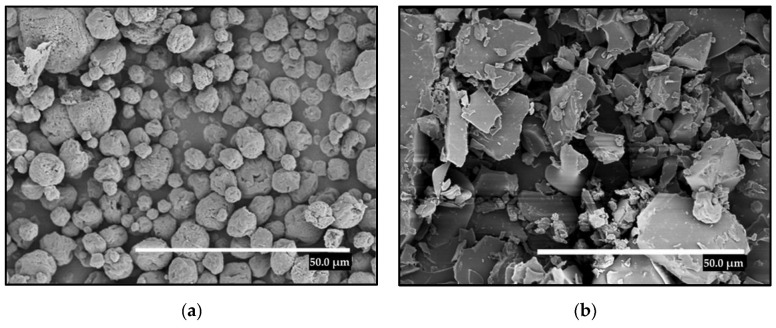
SEM micrographs of the dried DBS structures. (**a**) DBS microparticles obtained by the EAPG process. (**b**) DBS particles obtained by FD. The scale bar in both images corresponds to 50 µm.

Firstly, the EAPG and the FD processes were used to dry natural DBS. Figure 1 makes a comparison of the morphologies of the obtained particles with both technologies. By means of EAPG (Figure 1a), the production of individual spherical particles with an average particle size of 11.38 ± 4.34 μm, regular distribution, and without the presence of aggregates was achieved. These DBS microparticles presented some roughness with wrinkles and fissures on their surface.

As a comparison, natural DBS was also dried by FD. This technology was selected as a control technique since this method was reported as one of the most relevant processes for the preservation of heat-sensitive bioactive compounds of application in pharmaceuticals and foods [2]. DBS dried by FD exhibited irregular structures with a broad particle size distribution, as shown in Figure 1b. This broad size distribution could affect the dissolution rate, bioavailability, and also the organoleptic properties of the final product.

Among both techniques, EAPG provided smaller particles, with controlled size distribution and spherical morphology, which may enhance its dissolution rate when entering into contact with water and reduces or avoids the impact in the texture of a final product, for example in functional foods.

**Figure 2:**
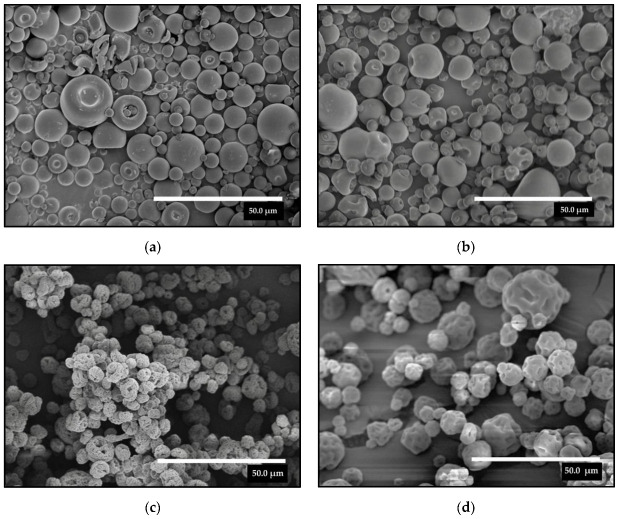
SEM micrographs of encapsulated DBS into WPC or ZN by the EAPG process. (**a**) WPC—DBS 1:1 *w*/*w*; (**b**) WPC—DBS 2:1 *w*/*w*; (**c**) ZN—DBS 1:1 *w*/*w*; (**d**) ZN—DBS 2:1 *w*/*w*. The scale bar in both images corresponds to 50 µm.

Secondly, WPC and ZN proteins were used to encapsulate natural DBS by the EAPG process in order to increase the stability of the DBS bioactive properties and functional capabilities. The morphology of the encapsulates of DBS into WPC and ZN at different encapsulant: bioactive ratios is shown in Figure 2. In all cases, spherical-like particles were obtained with uniform size distributions. Whereas particles obtained with WPC presented a smooth particle surface with the presence of some indentations, probably due to the high content of DBS in the microparticles, the particles obtained with ZN presented a rough surface, which is typical of this material, and it is produced due to the fast evaporation rate of the ethanol [33,34].

The structure of WPC was similar for the two ratios of DBS; however, in the case of ZN, by decreasing the amount of DBS in the particle, the roughness of the surface of the particles decreased. The particle size varied significantly depending on the encapsulating material used, between 6.37 to 12.77 μm as shown in Table 1, with it being smaller for the particles obtained with ZN. However, the increase in the amount of DBS did not result in a significant variation in the particle size with any encapsulating material.

### 2.2. Characterization of Polyphenol Content and Antioxidant Activity

Herein, the Folin–Ciocalteu method was used to analyze the total soluble polyphenols (TSP) of natural DBS, DBS dried by EAPG and FD, and the produced DBS encapsulates. The results obtained are shown in Table 1. No significant differences were observed between the natural DBS, the DBS dried by EAPG or FD, and the encapsulates, which means that the TSP was not affected during the process.

The antioxidant activity of these materials was also characterized via the DDPH method, and the results are also shown in Table 1. These results demonstrate that natural DBS has exceptionally high antioxidant activity, which correlates with the antioxidant activity of natural DBS reported by previous authors [35]. The antioxidant activity increased by drying the natural DBS by FD or EAPG in comparison to the natural DBS, probably due to humidity loss. However, no significant differences were observed between the drying process, EAPG or FD, which confirms that the EAPG method is a gentle method suitable for the processing of sensitive bioactive compounds.

However, regarding the encapsulates, it was possible to observe a slight reduction in antioxidant activity between the natural DBS, the dried DBS by EAPG or FD, and the encapsulates. This fact could also be produced by the weak DBS extraction from the produced WPC and ZN capsules. Similar results were obtained with both encapsulating materials. Nevertheless, whereas no significant differences were observed between the encapsulates produced with WPC at the studied ratios; in the case of ZN, higher antioxidant activity was observed for the ratio 2:1 *w*/*w* in comparison with the ratio 1:1 *w*/*w*.

### 2.3. Photo-Oxidation Stability

The photo-oxidation stability of the dried DBS samples and the encapsulates was first evaluated following the evolution of the TSP with time. The obtained results are shown in Table 2. The TSP for the dried DBS constantly decreased with time, but no significant differences were observed with the drying method since both drying methods showed similar decay rates.

According to the results shown in Table 2, both encapsulating materials provided a similar degree of protection, which could be related to the fact that the selected proteins are well-known for their capacity to block UV light and retard oxidation [36,37,38]. However, the ratio of encapsulant material: DBS seemed to be the most important factor. For instance, samples with a 2:1 *w*/*w* ratio presented a reduced TSP decay rate compared to examples of a 1:1 *w*/*w* ratio, which showed a similar decay rate to the dried DBS. This result could be due to a less efficient encapsulation process, as a consequence of the lack of enough encapsulating material to offer protection to the DBS present in the capsule.

In addition, a comparison of the photo-oxidation stability of the dried DBS samples and the encapsulation systems was performed in terms of the antioxidant activity measured via the DPPH method, and the results are shown in Table 3. According to the results, the dried DSB samples presented a significant decrease in DPPH inhibition with time, with no significant differences being observed in the decay rate between the drying methods. These results agree with the observations made of the TSP decay shown in Table 2. However, the DPPH inhibition values remained stable with time for the encapsulates, which could be related to the well-known capacity of WPC and ZN to block UV light and retard oxidation [36,37,38]. However, no significant differences were detected in the DPPH inhibition decay rate between these samples, neither between the proteins nor with the different ratios. In this sense, the Folin–Ciocalteu method allowed us to differentiate between the stability of the encapsulates in contrast with the DPPH method.

**Figure 3:**
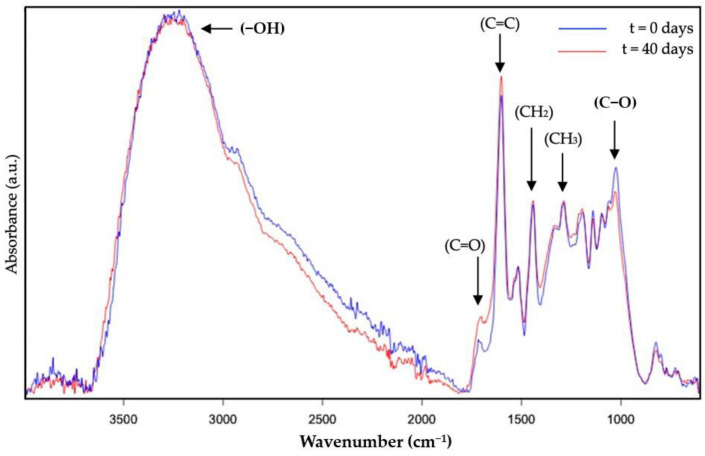
Attenuated total reflection—Fourier transform infrared spectroscopy (ATR- FTIR) spectral evolution over the accelerated oxidation test of DBS dried by EAPG. The spectra were normalized to the intensity of the internal standard band at ca. 1519 cm^−1^.

**Figure 4:**
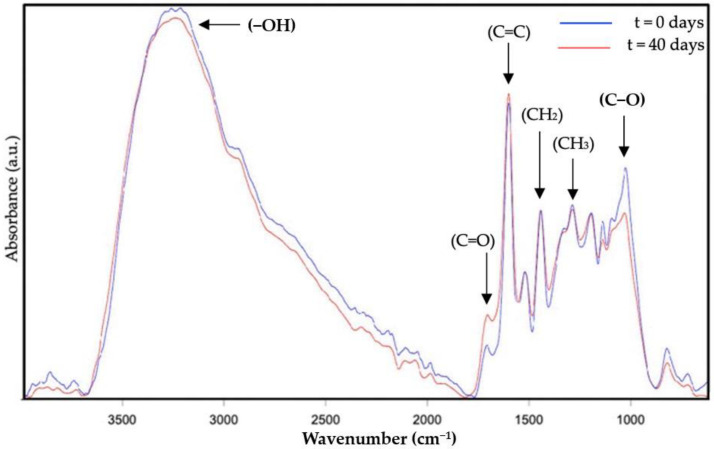
Attenuated total reflection—Fourier transform infrared spectroscopy (ATR- FTIR) spectral evolution over the accelerated oxidation test of DBS dried by freeze-drying (FD). The spectra were normalized to the intensity of the internal standard band at ca. 1519 cm^−1^.

The analysis of the photo-oxidation stability of the DBS samples was also performed by means of ATR-FTIR. Whereas Figure 3 represents the ATR-FTIR spectral evolution of the DBS dried by EAPG, Figure 4 represents the ATR-FTIR spectral evolution of the DBS dried by FD. Since the values of the intensity are arbitrary in the ATR-FTIR mode, to compare the results, the spectra were normalized to the intensity of the peak at ca. 1519 cm^−1^. This band was already used as an internal standard in our previous work [9], since it is assigned to the vibration of the C=C bond, typical of aromatic systems [39], and it did not show any variation during experimentation.

The band envelope between 3100 and 3600 cm^−1^ is associated with the stretching modes of the different -OH groups [40]. This band envelope reduced its intensity probably due to a loss of water as a consequence of the heat transferred by the lamp and due to the transformation of the hydroxyl group into the ether, aldehyde, or ketone groups as a consequence of the polyphenol oxidation reactions [41]. The following band at ca. 1780–1630 cm^−1^ is well defined, and it was associated with carbonyl stretching absorption [42], which is characteristic of the polyphenols present in natural DBS, such as taspin or epigallocatechin, and also the carbohydrates present in the sap, and it increases its intensity as oxidation proceeds because of the transformation of the alcohol group into aldehydes and ketone groups [41]. The bands at ca. 1600, 1530, and 1519 cm^−1^ are attributed to the stretching vibration of the aromatic alkenyl bond, which is also present in large amounts in polyphenols [43,44]. Only the band at 1600 cm^−1^ shows very little modification, less than 5%, which is probably influenced by the increased intensity of the carbonyl band. This band has not been selected as an internal standard, since it could be influenced by the humidity content of the samples, since one of the characteristic bands of water overlaps with this band. A peak around 1440 cm^−1^ was attributed to -CH deformation and aromatic ring vibration [44]. The peak around 1375 cm^−1^ was assigned to methyl bending [17]. The peaks between 1200 and 1070 cm^−1^ were due to C-O stretching in ether groups or aromatic alcohols [45], characteristics of polyphenols, and carbohydrates. This band decreased its intensity as a consequence of the polyphenol oxidation reactions, which transformed the aromatic alcohols and ether groups into ketones, carboxylic acids, or ester groups [46,47]. Finally, a band around 800 cm^−1^ was attributed to the out-of-plane C-H vibrations [45] and also to the humidity content of the sample, which decreased with time as explained before. Overall, these prominent characteristic peaks of the studied ATR-FTIR spectra agree with the bands previously reported for natural DBS [9].

The -OH, C=O, and C-O characteristic bands were found to provide the most significant contribution to the oxidation of polyphenols [47]; however, only the C=O and the C-O characteristic bands were selected as the most representative to perform the comparison of the dried DBS samples in terms of photo-oxidation stability since the -OH group could be affected by the sample’s humidity.

**Figure 5:**
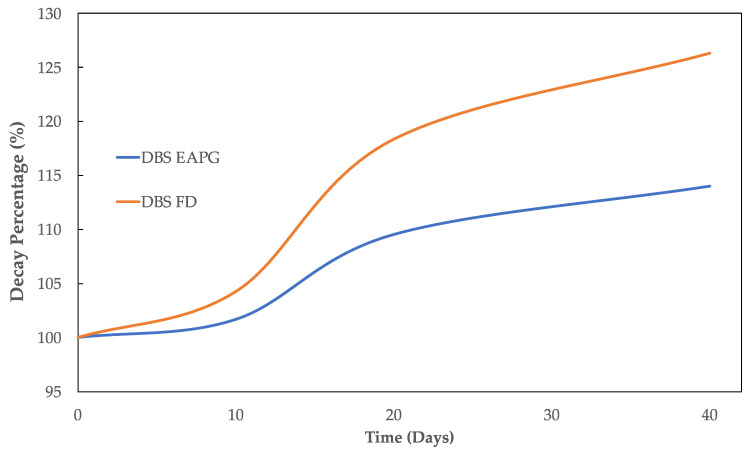
Evolution of the decay percentage of the DBS characteristic carbonyl functional group (C=O) (1715 cm^−1^) normalized to the standard internal band at ca. 1519 cm^−1^ for the DBS dried by EAPG and by freeze-drying (FD) during 40 days under UV light radiation.

Figure 5 represents the evolution of the decay percentage of carbonyl group intensity normalized to the standard internal peak at ca. 1519 cm^−1^ of the dried DBS samples. The increase in the intensity of the carbonyl compounds could be generated from the oxidation reactions due to the formation of ester, carboxylic acids, and ketone groups [46]. The increase was a little bit more intense for the FD sample.

**Figure 6:**
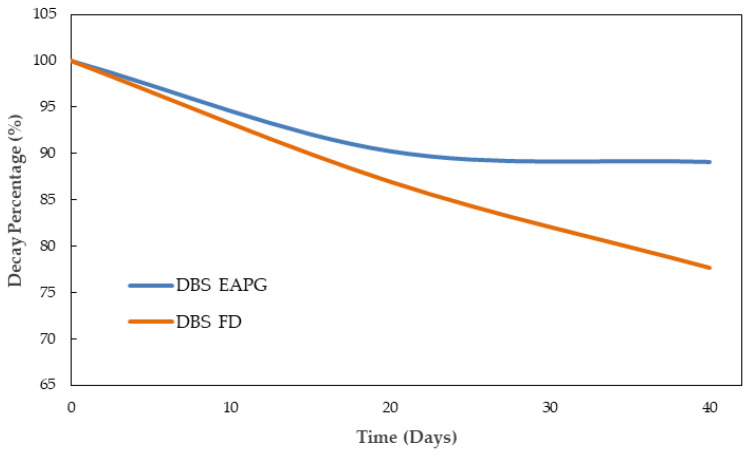
Evolution of the decay percentage of the DBS characteristic C-O band (1070 cm^−1^) normalized to the standard internal band at ca. 1519 cm^−1^ for the DBS dried by EAPG and by freeze-drying (FD) during 40 days under UV light radiation.

Figure 6 represents the evolution of the decay percentage of the C-O band normalized to the standard internal band at ca. 1519 cm^−1^ for the dried DBS samples. This band decreased its intensity as a consequence of the polyphenol oxidation reactions, which transformed the aromatic alcohols and ether groups into ketones, carboxylic acids, or ester groups [46,47]. Despite both samples showing a similar decay rate during the first 20 days, after this point, the decay of the C-O band remained stable for the EAPG processed sample, whereas it continued to decrease for the freeze-dried sample.

Although no significant differences were observed between these two drying methods, the obtained results agree with the previous analysis performed in terms of TSP and DPPH inhibition and allowed us to prove that by using the EAPG process, there were no significant chemical alterations in this bioactive ingredient in comparison to FD.

**Figure 7:**
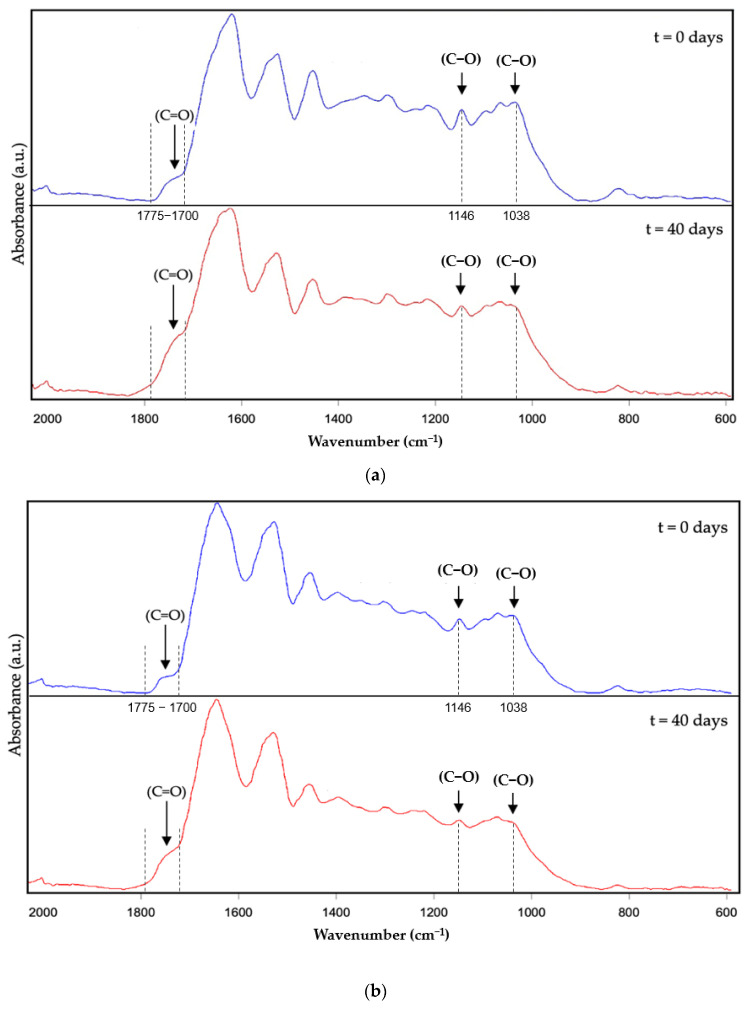

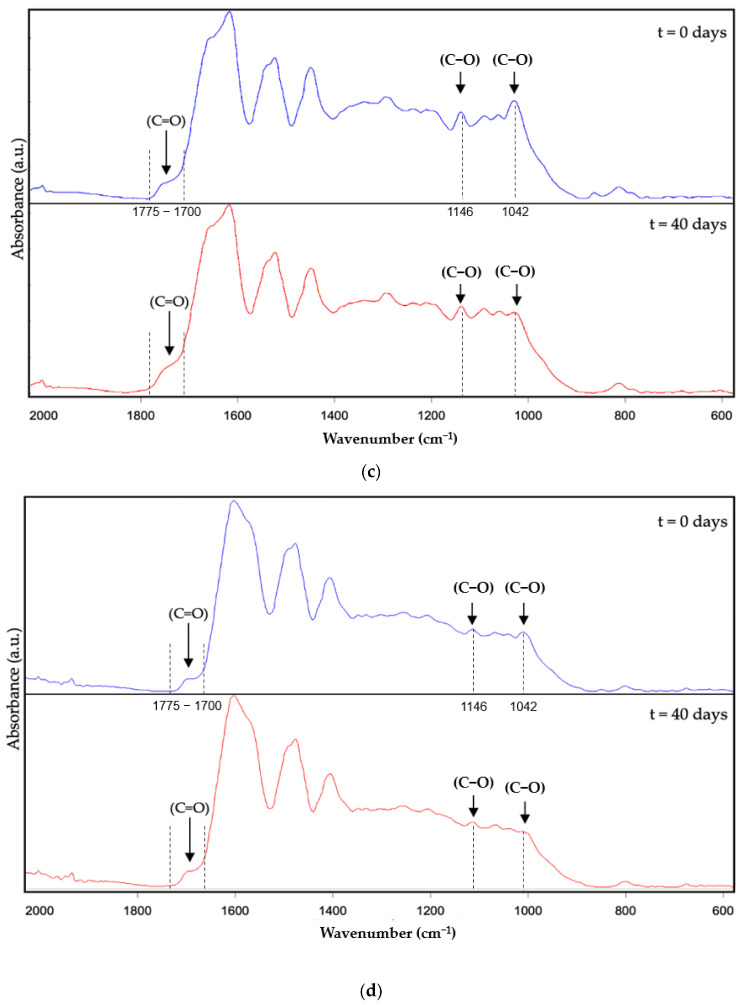
Attenuated total reflection—Fourier transform infrared spectroscopy (ATR- FTIR) spectral evolution over the accelerated oxidation test. (**a**) WPC—DBS 1:1 *w*/*w*, (**b**) WPC-DBS 2:1 *w*/*w*, (**c**) ZN-DBS 1:1 *w*/*w*, (**d**) ZN-DBS 2:1 *w*/*w*. The spectra were normalized to the highest intensity band in the wavelength range between 2000 and 600 cm^−1^.

Figure 7 shows the ATR-FTIR spectral evolution of protein: DBS encapsulates after 40 days of the accelerated oxidation test. Herein, the most significant spectral changes regarding DBS oxidation occurred in the wavenumber range between 2000 and 600 cm^−1^, and for comparison purposes, the spectra were maximized to the highest intensity band in this wavenumber range.

The main characteristic bands of WPC (spectrum not shown) were located at ca. 1740 cm^−1^ ascribed to the stretching vibration of the C=O bond, at ca. 1632 cm^−1^ and ca. 1520 cm^−1^ ascribed to the bending vibration of the N-H bond of amides I and II, respectively, two bands at ca. 1448 and 1391 cm^−1^ ascribed to the bending vibration of the -CH bond, a band at ca. 1237 cm^−1^ ascribed to the axial deformation vibrations of the C-N bond, and two bands at 1153 and 1076 cm^−1^ ascribed to the stretching vibration of the C-O bond [48,49]. Regarding the ZN (spectrum not shown), the main characteristic bands were located at ca. 1735 cm^−1^ ascribed to the stretching vibration of the C=O bond, the bands at ca. 1642 cm^−1^ and at ca. 1517 cm^−1^ ascribed to the bending vibration of the N-H bond of amides I and II, respectively, a band at ca. 1447 cm^−1^ assigned to the bending vibration of the -CH bond, a band at ca. 1235 cm^−1^ ascribed to the axial deformation vibrations of the C-N bond, and a band at ca. 1168 cm^−1^ ascribed to the stretching vibration of the C-O bond [50]. Concerning the DBS, the main characteristic bands have been already described.

The characteristic bands of the DBS and proteins were observed in all the encapsulated ATR-FTIR spectra shown in Figure 7. The encapsulates of WPC-DBS 1:1 *w*/*w* and 2:1 *w*/*w*, shown in Figure 7a,b, showed a band envelope between 1775 and 1700 cm^−1^, which is the result of the overlapping of the contributions of the stretching vibration of the C=O bonds of the protein and the C=O bonds of the DBS, the band envelope between 1700 and 1568 cm^−1^ which is the result of the overlapping of the band ascribed to the bending vibration of the N-H bond of amide I of the protein and the band ascribed to the C=C bond of the DBS, the band envelope between 1568 and 1482 cm^−1^, which is the result of the overlapping of the contribution of the C=C bond and the bending vibration of the N-H bond of amide II, three bands at ca. 1445, 1388, and 1340 cm^−1^ ascribed to the bending vibration of the CH bond, and a band envelope between 1300 and 1000 cm^−1^, which could be ascribed to the stretching vibration of the C-O bond. The band at ca. 828 cm^−1^ is attributed to the out-of-plane C-H vibrations and also the humidity content of the sample.

The encapsulates of ZN-DBS 1:1 *w*/*w* and 2:1 *w*/*w*, shown in Figure 7c,d, showed a characteristic band envelope between 1775 and 1700 cm^−1^, which is the result of the overlapping of the contributions of the stretching vibration of the C=O bonds of the protein and the C=O bonds of the DBS, the band envelope between 1700 and 1568 cm^−1^, which is the result of the overlapping of the band ascribed to the bending vibration of the N-H bond of amide I and the band ascribed to the C=C bond, the band envelope between 1568 and 1480 cm^−1^, which is the result of the overlapping of the contribution of the C=C bond and the bending vibration of the N-H bond of amide II, three bands at ca. 1444, 1368, and 1339 cm^−1^ assigned to the bending vibration of the CH bond, and a band envelope between 1300 and 1000 cm^−1^, which could be ascribed to the stretching vibration of the C-O bond. The band at ca. 877 cm^−1^ may be due to the ethanol residual content in the particles, which disappears within 40 days with the heat provided by the lamp. The band at ca. 828 cm^−1^ is attributed to the out-of-plane C-H vibrations and also the humidity content of the sample.

As shown in Figure 7, the most significant changes were identified in the band envelope between 1775–1700 cm^−1^, associated with the carbonyl stretching absorption, highly present in polyphenols, proteins, and carbohydrates and in the band envelope at ca. 1300–1000 cm^−1^ ascribed to the stretching vibration of the C-O bond of not only aromatic alcohols, ethers, and esters groups of polyphenols, but also present in carbohydrates. Whereas the carbonyl envelope increased its relative intensity with UV time exposure, some bands in the C-O bond envelope decreased their relative intensity with UV time exposure. This occurred because of the oxidation reactions of polyphenols, which transformed the aromatic alcohols and ether groups into ketones, carboxylic acids, or ester groups [46,47]. However, the variations in the relative intensity of these band envelopes were less pronounced than in the dried DBS achieved by EAPG (Figure 3), thanks to the protection offered by the proteins.

In particular, the encapsulates obtained using WPC in both encapsulation ratios, 1:1 *w*/*w* and in 2:1 *w*/*w* (Figure 7a,b), resulted in clear alterations in the bands at ca. 1146 cm^−1^ and 1038 cm^−1^ and also in the relative intensity of the band envelope between 1775–1700 cm^−1^. The cited spectral variations were more pronounced in the 1:1 *w*/*w*, since there is a lower amount of protein with respect to the DBS to offer UV protection. In the case of the capsules obtained using ZN (Figure 7c,d), alterations in the relative intensity of the bands at ca. 1146 and 1042 cm^−1^ and in the band envelope between 1775 and 1700 cm^−1^ were observed with UV light exposure, similar to how it was observed for the WPC encapsulates. For this protein, also in a 1:1 *w*/*w* ratio, the most pronounced variation in the relative intensity of the cited bands was shown. The tendency observed in these bands agrees with the TSP results obtained for the encapsulates shown in Table 2. However, among the two proteins at a ratio of 2:1 *w*/*w*, ZN showed the lowest spectral variations in both band envelopes, and consequently, this formulation was proposed as the most stable.

## 3. Materials and Methods

The dragon’s blood sap (DBS) was provided by Q’omer BioActive Ingredients (Valencia, Spain). The natural DBS was used as received without any further purification. Whey protein concentrate (WPC) 80% was purchased from Beurrespa (Madrid, Spain). According to the distributor, the WPC was said to contain 81.6% protein (on a dry basis), 7.5% fat, and 4.3% moisture. The maize zein (ZN) protein, gallic acid, sodium carbonate, Folin–Ciocalteu reagent, and Span 20 were purchased from Sigma-Aldrich (Saint Louis, MO, USA). The methanol (reagent grade) was purchased from Labkem-Labbox (Mataró, Spain). Ethanol 96% (*v*/*v*) was purchased from Panreac Química SLU (Barcelona, Spain). Deionized water was used throughout the study.

### 3.1. Determination of Total Solids

The total solids content was determined by measuring the mass of the sample of DBS before and after the water was removed. The evaporation process was performed at 60 °C using a vacuum oven (Vaciotem-T, JP. Selecta, Barcelona, Spain) for 48 h. The total solid content was calculated according to Equation (1):

Total Solids (%) = (M dried/M initial) × 100(1)

where M dried is the final sample weight and M initial is the initial sample weight.

### 3.2. Solution Preparation

First, a solution for the encapsulating materials was prepared. WPC was dissolved into water at 20% (*w*/*v*) concentration and ZN was dissolved into 85% (*v*/*v*) ethanol aqueous solution at 4% (*w*/*v*) concentration. Then, natural DBS resin was slowly added to the corresponding encapsulant solution into a 1:1 *w*/*w* and a 2:1 *w*/*w* encapsulant to DBS ratio. The 2:1 *w*/*w* ratio was selected according to our previous experience in the encapsulation of bioactive compounds [24]; however, since water in DBS evaporates during the EAPG process, an increased content of DBS in the particle was also considered, concretely the ratio 1:1 *w*/*w*. These ratios were calculated between the amount of encapsulating material and the solid content of DBS resin determined according to Section 3.1 (natural resin contains around 26% solids (wet basis)). The total solids content in the solution for the WPC formulations was 22% for the ratio of 1:1 and 21% for the ratio of 2:1, and for the ZN formulations, this was 7% for the ratio of 1:1 and 5% for the ratio of 2:1.

The solution was homogenized using a magnetic stirrer (H20 series, LBX Instruments, Premia de Dalt, Spain) at ambient temperature overnight until total DBS dissolution.

### 3.3. EAPG Process

First, the natural DBS was dried by EAPG using the proprietary Capsultek^TM^ pilot plant from Bioinicia S.L. (Valencia, Spain). This pilot installation comprises a nebulizer, the products of which are subjected to an electric field, a drying chamber, and a cyclonic collector, as described by Busolo et al. [28]. The experiments were developed at controlled ambient conditions, 25 °C and 30% relative humidity (RH). The DBS was pumped at a flow rate of 10 mL/h into the injection unit, with an air pressure of 10 L/min and a voltage of 15 kV. Particles were collected from the cyclone, stored in flasks, under vacuum, at −20 °C, and protected from light to avoid oxidation until further analysis.

Secondly, the encapsulation of DBS in the selected encapsulation materials via EAPG was performed by processing previously prepared solutions using the same process parameters.

For these solutions and with this pilot installation, the efficiency of the process was around 50%; however, it can increase up to 90–100% at an industrial scale.

### 3.4. Freeze-Drying (FD)

The natural DBS was also processed by FD to compare the EAPG drying process with conventional drying technology. This comparison allows comparing the obtained materials in terms of morphology, bioactive properties, and stability. Pure DBS was frozen at −80 °C in a static stage for two days and then dried on VirTis Genesis 35 EL freeze-drier (SP Scientifics, New York, NY, USA) equipment with a condenser temperature of −50 °C and a chamber pressure *p* < 0.08 mbar for 48 h. The FD samples were stored in flasks under vacuum at −20 °C and protected from light to avoid oxidation until further analysis.

### 3.5. Humidity

The humidity of the samples was calculated by drying the samples until obtaining a constant weight at 40 °C and by using a vacuum oven (Vaciotem-T, J.P. Selecta, Barcelona, Spain). The humidity content was determined from the difference in weight before and after drying.

The humidity contained in the dried samples was 4.7 ± 2.9% and 9.3 ± 1.5% for EAPG and FD, respectively.

### 3.6. Microscopy

Scanning electron microscopy (SEM) in a Hitachi S-4800 FE-SEM (Hitachi High Technologies Corp., Tokyo, Japan) was used to analyze the particles’ morphology. The microscope was settled with an electron beam acceleration of 5 kV. Approximately 5 mg of capsules of each sample were coated with a gold/palladium layer for SEM analysis. The average diameters were determined using Image J Launcher v1.41 (National Institutes of Health, Bethesda, MD, USA).

### 3.7. Photo-Oxidation Stability of the DBS Capsules

The particles encapsulating the DBS were exposed to ultraviolet (UV) radiation to accelerate DBS oxidation. An OSRAM Ultra-Vitalux lamp (OSRAM, Garching, Germany) irradiated the samples with UV light. A blend of radiation, similar to natural sunlight, is generated by a quartz discharge tube and a tungsten filament, operating the lamp with a power of 300 W. Only similar wavelengths to that of daylight pass through the special glass bulb. The radiation between 315–400 nm after 1 h of exposure is 13.6 W, and the radiation between 280 and 315 nm after 1 h of exposure is 3 W.

The photo-oxidation stability assay was developed at ambient temperature under UV light for 40 days. Approximately 20 g of the capsules was placed onto Petri dishes at 20 cm under a UV lamp. The thickness of the powder in the Petri dish was lower than 5 mm. To ensure homogenous treatment, the powder was stirred on a daily basis. Photo-oxidation was monitored by evaluating total soluble polyphenols, antioxidant capacity, and ATR-FTIR spectroscopy.

### 3.8. Total Soluble Polyphenols

The Folin–Ciocalteu method was used to analyze the total soluble polyphenols (TSP) of natural DBS, dried DBS, and DBS-loaded capsules. Approximately 10 mg of DBS and the equivalent amount of capsules were diluted in ethanol at 70% (*v*/*v*). After dissolution, 20 μL of the capsule’s solution was mixed with 1.2 mL of water and 300 μL of sodium carbonate 7.5% (*w*/*v*) in Eppendorf tubes and homogenized for 1 min in a vortex shaker and left to stand for 5 min. Subsequently, it was mixed with 380 μL of water, and then 100 μL of the Folin reagent solution 10% (*v*/*v*) was added to react for 15 min in dark conditions and at room temperature. Absorbance was measured in triplicate at 765 nm using a UV/V is spectrophotometer (UV4000 Dinko Instruments, Barcelona, Spain). The results were expressed in gallic acid equivalents (mg GAE/g of natural DBS).

### 3.9. Antioxidant Activity

The DPPH free radical scavenging method was used to analyze the antioxidant capacity of the DBS capsules [2]. Approximately 10 mg of DBS and the equivalent amount of capsules were dissolved in 1 mL of methanol. Thereafter, 50 μL of the capsule’s solution was dissolved in 950 μL of methanol. An aliquot of 0.1 mL of DBS methanol solution (0.5 mg/mL) was added to 1.9 mL of DPPH methanolic solution (0.094 mM), vortexed, and kept in dark conditions for 30 min at room temperature. The methanol solution was used as a blank, and absorbance was measured in triplicate at 517 nm using a UV/Vis spectrophotometer (UV4000 Dinko Instruments, Barcelona, Spain). The radical scavenging activity was calculated according to Equation (2):

IP-DPPH (%) = ((Ac − As)/Ac) × 100(2)

where IP-DPPH (%) is the inhibition percentage of DPPH, Ac is the absorbance of pure DPPH solution, and As is the absorbance of the incubated sample after reacting with DPPH.

### 3.10. Attenuated Total Reflection–Fourier Transform Infrared (ATR-FTIR)

A Bruker Tensor 37 FT-IR Spectrometer (Bruker, Ettlingen, Germany) was used to measure the ATR-FTIR spectra of DBS capsules. The low-temperature ATR sampling accessory Golden Gate (Specac Ltd., Orpington, UK) was used to guarantee proper contact between the diamond crystal and DBS encapsulated samples (50 mg approximately). All spectra were recorded within the wavenumber range 4000–600 cm^−1^ by averaging 10 scans, with a resolution of 4 cm^−1^. The results were normalized to the intensity of the band at 1519 cm^−1^ for better comparison among the different capsules. Analysis of spectral data was carried out using the OPUS 4.0 data collection software program (Bruker, Ettlingen, Germany). The measurements were performed in triplicate.

### 3.11. Statistical Analysis

Statgraphics Centurion Version 17.2.04 (Statistical Graphics Corp., Warrenton, VA, USA) was used for the data analysis. The data were expressed as the mean ± standard deviation. The significance of the results obtained for the different samples was studied by analysis of variance (ANOVA) followed by Tukey’s test. Differences were considered significant at *p*-value < 0.05.

## 4. Conclusions

In this work, the electrospraying assisted by pressurized gas (EAPG) method was used for the first time to dry DBS, and the characteristics of the obtained dried particles were compared with the dried DBS particles obtained by FD, a reference technique for the drying of sensitive bioactive compounds. Both techniques produced dried DBS particles with similar TSP and antioxidant properties, and similar photo-oxidation stability. According to the ATR-FTIR results, polyphenols oxidation reactions took place during the photo-oxidation test, transforming the aromatic alcohols and ether groups, into ketones, carboxylic acids, or ester groups, which could be the reason for the loss in antioxidant activity. However, whereas EAPG produced DBS spherical microparticles with controlled particle size distribution, FD produced irregular particles with a broad particle size distribution, which could affect the dissolution rate, the bioavailability, and the organoleptic impact in a final food product. Secondly, the encapsulation of DBS into protein-based systems, WPC and ZN, was successfully achieved for the first time via EAPG, with it obtaining free-flowing powder with controlled particle size. This encapsulation process contributed to protecting the DBS polyphenolic compounds without affecting their antioxidant bioactivity and TSP. Moreover, it was possible to protect the DBS from UV light radiation, increasing the stability of this bioactive ingredient, especially when using ZN as an encapsulating material in the ratio 2:1 *w*/*w*, since the minimum chemical alterations in the ATR-FTIR bands related to polyphenol oxidation were detected. The obtained results showed that this novel process offers an innovative mild encapsulation and drying technology with the advantage, compared to, for instance, FD, in that it is a continuous process available at an industrial scale. In conclusion, considering the obtained results, these microparticles could be promoted as functional food ingredients adequate to be used in the formulation of functional foods, nutraceuticals, nutracosmetics, or pharmaceuticals, among others.

## Figures and Tables

**Table 1:** Particle size, total soluble polyphenol content, and antioxidant activity for DBS dried by FD (DBS-FD), DBS dried by EAPG (DBS-EAPG), and encapsulated DBS into whey protein concentrate (WPC) and zein (ZN).

Sample	Particle Size (μm)	TSP (mg GAE/g Natural DBS)	IP-DPPH (%)
Natural DBS	-	2.05 ± 0.03 ^a^	93.39 ± 0.01 ^a^
DBS—FD	-	2.06 ± 0.07 ^a^	96.02 ± 0.04 ^b^
DBS—EAPG	11.38 ± 4.34 ^a^	2.03 ± 0.06 ^a^	95.27 ± 0.06 ^b^
WPC—DBS 1:1	11.28 ± 4.28 ^a^	2.07 ± 0.08 ^a^	80.29 ± 0.02 ^cd^
WPC—DBS 2:1	12.77 ± 4.54 ^a^	2.03 ± 0.05 ^a^	79.88 ± 0.02 ^d^
ZN—DBS 1:1	6.37 ± 1.67 ^b^	2.01 ± 0.12 ^a^	79.64 ± 0.03 ^d^
ZN—DBS 2:1	7.58 ± 2.54 ^b^	2.07 ± 0.11 ^a^	82.94 ± 0.01 ^c^

Mean values ± standard deviation with different superscripts in the same column are significantly different (Tukey’s test, *p* ≤ 0.05). TSP—total soluble polyphenols. GAE—gallic acid equivalent. IP-DPPH—inhibition percentage of the DPPH reactive.

**Table 2:** Evolution of the TSP of dried DBS by EAPG and freeze-drying (FD), and the encapsulated DBS into WPC and ZN protein at 1:1 *w*/*w* and 2:1 *w*/*w* ratios over a period time of 40 days under UV light exposure.

Sample	TSP Decay (%)		
	UV Light Exposure Time (Days)		
	0	20	40
DBS-FD	100	78.44 ± 4.97 ^d^	59.19 ± 6.78 ^e^
DBS-EAPG	100	85.41 ± 2.11 ^bc^	61.69 ± 7.56 ^e^
WPC-DBS 1:1 *w*/*w*	100	77.01 ± 1.23 ^d^	60.87 ± 8.23 ^e^
WPC-DBS 2:1 *w*/*w*	100	94.54 ± 2.13 ^a^	81.69 ± 4.76 ^ab^
ZN-DBS 1:1 *w*/*w*	100	80.12 ± 5.15 ^bcd^	63.34 ± 2.34 ^e^
ZN-DBS 2:1 *w*/*w*	100	94.27 ± 1.57 ^a^	91.92 ± 6.47 ^ab^

Mean values ± standard deviation with different superscripts in the same column are significantly different (Tukey’s test, *p* ≤ 0.05).

**Table 3:** Evolution of the antioxidant activity measured as DPPH inhibition percentage of dried DBS by EAPG and freeze-drying (FD) and encapsulated systems of DBS into WPC and ZN at 1:1 *w*/*w* and 2:1 *w*/*w* ratio over a period time of 40 days under UV light exposure.

Sample	DPPH Inhibition Decay (%)		
	UV Light Exposure Time (Days)		
	0	20	40
DBS-FD	100	87.92 ± 1.65 ^b^	70.07 ± 0.39 ^c^
DBS-EAPG	100	87.84 ± 3.34 ^b^	72.56 ± 0.80 ^d^
WPC-DBS 1:1 *w*/*w*	100	99.62 ± 2.78 ^a^	98.79 ± 4.88 ^a^
WPC-DBS 2:1 *w*/*w*	100	100.28 ± 4.85 ^a^	98.35 ± 3.44 ^a^
ZN-DBS 1:1 *w*/*w*	100	98.63 ± 3.66 ^a^	99.76 ± 3.11 ^a^
ZN-DBS 2:1 *w*/*w*	100	99.50 ± 2.10 ^a^	98.92 ± 2.20 ^a^

Mean values ± standard deviation with different superscripts in the same column are significantly different (Tukey’s test, *p* ≤ 0.05).

## Data Availability

Data is contained within the article.

## Sample Availability

Samples of the compounds could be available from the authors on request.
